# Recovery of Residual Lead from Automotive Battery Recycling Slag Using Deep Eutectic Solvents

**DOI:** 10.3390/molecules29020394

**Published:** 2024-01-13

**Authors:** Bruna Salgado, Diana Endara, Carlos F. Aragón-Tobar, Ernesto de la Torre, Luis Ullauri

**Affiliations:** Department of Extractive Metallurgy, Escuela Politécnica Nacional, Ladrón de Guevara E11-253, P.O. Box 17-01-2759, Quito 170525, Ecuador; diana.endara@epn.edu.ec (D.E.); carlos.aragont@epn.edu.ec (C.F.A.-T.); ernesto.delatorre@epn.edu.ec (E.d.l.T.); luis.ullauri@epn.edu.ec (L.U.)

**Keywords:** foundry slag, automotive battery recycling, lead, deep eutectic solvents

## Abstract

In this study, we address the ecological challenges posed by automotive battery recycling, a process notorious for its environmental impact due to the buildup of hazardous waste like foundry slag. We propose a relatively cheap and safe solution for lead removal and recovery from samples of this type of slag. The analysis of TCLP extracts revealed non-compliance with international regulations, showing lead concentrations of up to 5.4% primarily in the form of anglesite (PbSO_4_), as detected by XRF/XRD. We employed deep eutectic solvents (DES) as leaching agents known for their biodegradability and safety in hydrometallurgical processing. Five operational variables were systematically evaluated: sample type, solvent, concentration, temperature, and time. Using a solvent composed of choline chloride and glycerin in a 2:1 molar ratio, we achieved 95% lead dissolution from acidic samples at 90 °C, with agitation at 470 rpm, a pulp concentration of 5%, and a 5 h duration. Furthermore, we successfully recovered 55% of the lead in an optimized solution using an electrowinning cell. This research demonstrates the ability of DES to decontaminate slag, enabling compliance with regulations, the recovery of valuable metals, and new possibilities for the remaining material.

## 1. Introduction

Annually, over 9 million tons of lead are estimated to be produced, with approximately 86% of this supply dedicated to manufacturing electrodes for lead-acid Batteries (LABs), which serve as the primary source for starting vehicle engines [1]. While these batteries are essential devices, they also pose a significant hazard due to their toxic components [2]. Every year, millions of batteries reach the end of their life cycle, and disposing of them in regular garbage dumps or sanitary landfills is not a viable option. Consequently, more than 85% of them need to be recycled. Furthermore, over half of the world’s lead production comes from secondary sources, which consume less energy compared to primary extraction from ores [3]. However, these secondary processes are not exempt from having an intense environmental impact [4]. According to Pure Earth (2016), the recycling of LABs is listed as one of the top ten most polluting industrial processes [5].

Battery recycling plants generally operate through pyrometallurgical extraction. This process involves the removal of lead residues from plastic cases and their placement on metallurgical furnaces where carbon-based reduction occurs, aided by fluxing and slagging agents [6]. For every ton of lead produced, 300–350 kg of slag is generated [7], which is often stacked in the open air, lacking proper treatments [8]. This residue is a by-product whose composition depends on operating variables such as furnace temperature, heat transfer efficiency, and atmospheric pressure, as well as the additives that are incorporated into the mixture. Despite extensive reprocessing efforts, some lead will always remain in the residue. This toxic and persistent metal poses contamination risks for air, soil, and water [6]. Continuous human exposure to this metal can be prejudicial to health, leading to kidney, liver, and nervous system failure. Lead can also infiltrate plant roots, making its way into the food chain and posing a threat to all life forms [9]. The substantial amount of slag produced introduces heavy metals into the environment and represents a significant loss of resources. Therefore, it is crucial to explore sustainable alternatives to recover the lead stored in the slag, while simultaneously decontaminating it for a safe final disposal.

Many studies focus on developing stabilization/solidification (S/S) processes to physically encapsulate the slag within building materials such as geopolymers, concrete aggregates, asphalt mixtures, refractories, and ceramics [8]. The objective of S/S is to reduce the chemical reactivity of the slag while taking advantage of its mechanical properties. However, there is still a risk of natural leaching of toxic elements, underscoring the need for initial heavy metal removal. Although lead has a low leaching rate in weak acid environments, prolonged exposure to water can result in a significant release of this element [10].

There are three lead recovery methods that have been studied. First is pyrometallurgy, which involves reprocessing within smelting furnaces. However, it generates a larger volume of slag and requires elevated energy consumption. Also, given the great presence of sulfides on the secondary slag, pyrometallurgical reprocessing may be unsuitable for lead recuperation [10]. The second method involves bioleaching, where degrading microorganisms are used to convert metal compounds into recoverable, soluble, and extractable forms [11]. Unfortunately, this process is still currently limited to the laboratory scale. Finally, there is hydrometallurgy, where leachants are used to dissolve metal compounds, which are then recovered by electrodeposition or solvent extraction [10].

The typical hydrometallurgical route for lead recovery from battery scraps involves leaching with hydrofluorosilicic acid (H_2_SiF_6_). It has been demonstrated that conducting an electrowinning process in this solvent leads to the recovery of nearly 99% of the antimony-free lead at the cathode [12]. Sadly, many commonly used solvents are not environmentally friendly and may possess a considerable degree of toxicity.

Recently, ion metallurgy, a new branch of hydrometallurgy has emerged replacing traditional solvents with ionic liquids (IL) and molten salts [13]. Ionic liquids are entirely composed of ions with a hybrid organic-ionic nature and melting points below 100 °C. There are numerous combinations of cations and anions whose diverse behaviors can be useful for multiple applications [14]. Preliminary studies have demonstrated the potential of ILs as solvents for extracting gold from minerals [15] and for recovering uranium and plutonium from spent nuclear fuel [13]. Regarding lead, Tan et al. (2021) evaluated the use of N,N,N Dimethylbutylammonium Methanesulfonate as an electrolyte for the extraction and deposition of Pb from LABs. The electrochemical kinetics of lead reduction showed fast deposition and the possibility of up-scaling the entire process for industrial use [16]. However, it is worth noting that ILs have drawbacks, including a high cost, low availability, sensitivity to moisture, and the requirement of an inert environment during electrodeposition [17].

As an alternative, deep eutectic solvents (DES) have gained attention for their dissolution properties, which are similar to ILs but are easier to synthesize and use [17]. Unlike ILs, they are not entirely composed of ionic species. DES are typically composed of two or three components in specific proportions that can form eutectic mixtures through hydrogen bond interaction. These eutectic mixtures are characterized by having lower melting points than that of each individual component, usually below 70 °C [18].

Most of the compounds that constitute DES are safe, biodegradable, and increasingly available [19]. Key characteristics of deep eutectic solvents include elevated viscosity, high surface tension [20], low melting points, solubility and chemical inertness in water, and relative easiness to synthetize [21]; they are also safe, stable, non-volatile, and non-flammable [18]. DES are versatile in their ability to donate or accept electrons or protons and form hydrogen bonds, which gives them an anionic character and excellent dissolution capacities. They can be tunable for specific physical or chemical requirements depending on the metal of interest. A working principle is that DES consist of the available protons acting as oxygen acceptors and breaking the metal-oxygen bonds. Once the oxide is protonated, intermediate species are formed and can act as active sites for ligand complexation [22]. Another theory has been described where oxygen remains attached to the metal center and the hydrogen bond donors act as ligands [23].

Solubility can be influenced by pH; more acidic DES are better solvents due to higher H^+^ activity. However, solubility is also affected by the Gibbs energy, formation energy, and lattice energy of the metal oxide that must be overcome. Other factors such as changes in speciation and the coordination number of the metal cation in the produced intermediate compound also affect solubility [22]. Temperature is another solubility factor because as it increases, pH decreases linearly [24]. High temperatures also diminish the viscosity of the solvent, which can elevate the reaction rate and the mobility of species [18].

Typically, the quaternary ammonium salt used for the preparation of deep eutectic solvents is choline chloride (ChCl), a readily available, cost-effective, and safe compound [18]. The hydrogen bond donors that were investigated in the present study include Urea and renewable polyols such as Ethylene Glycol and Glycerol. ChCl-Urea is also known as reline, ChCl-Ethylene Glycol is known as ethaline, and ChCl-Glycerol goes by the name of glyceline. The selected DES’s physical properties are summarized in Table 1.

Before delving into the potential applications of DES, it is important to acknowledge that, despite their green solvent classification, they are not exempt from causing environmental issues. While deep eutectic solvents exhibit non-toxicity and favorable biocompatibility, the potential for long-term toxicity and bio-incompatibility has not received sufficient attention yet. Exposure through contact, inhalation, or accidental ingestion poses a threat to both cellular and noncellular life. Additionally, the processes of hydrolysis, decomposition, volatility, combustion, and flammability can generate toxic byproducts. The absence of comprehensive toxicological and biodegradable data could prevent industrial applications from becoming widespread [29,30]. When considering air pollution, a significant impact is evident when the chemical compounds utilized in the synthesis exhibit a volatile or semi-volatile nature. It is apparent that, under dynamic conditions, gas–liquid equilibrium may lead to the partial evaporation of these compounds [31]. When assessing the impact on aquatic environments, it is important to note that high water miscibility and solubility in aqueous solutions can pose a potential threat to bodies of water and the organisms inhabiting them. In this context, it is crucial to explore degradation techniques, such as enzymatic or oxidation methods, to selectively remove or decompose these substances [32].

In the context of employing deep eutectic solvents for metal ion extraction, some applications have already been explored. For instance, Haq et al. (2021) introduced and validated a novel methodology for zinc extraction. This method was specifically designed for the analysis of this metal in real fish and eel samples using Flame Atomic Absorption Spectroscopy (FAAS). The approached method demonstrated excellent precision and high recovery rates, and it achieved a quantification limit as low as 0.136 µg/kg [33]. Through the impregnation of silver nanoparticles into deep eutectic solvents (DES), researchers successfully developed a rapid ultrasonic-assisted microextraction system for the determination of lead (II) in edible oil samples. Following the optimization of validation parameters, an impressive quantification limit as low as 0.94 µg/L was attained. This achievement underscores the expanding influence of deep eutectic solvents in analytical chemistry [34]. But DES are also starting to expand their applications in the field of metallurgy.

One study demonstrated the applicability of the ChCl-Urea system to various lead compounds, including PbSO_4_, PbO_2_, and PbO, all of which exhibited similar solubility and electrochemical behavior [2]. Despite the fact that very little information has been found regarding the electrowinning of Pb from DES, there are studies where deep eutectic solvents have been applied in the electrolytic recovery of other metals. For instance, reline has been used for the dissolution and electrowinning of zinc from electric arc furnace dust. Cyclic voltammetry revealed cathodic sweeps with two deposition peaks, one at −0.46 V corresponding to lead impurities and another at −1.56 V corresponding to zinc. The study concluded that the decontamination of the dust was possible and that the impure zinc-lead obtained could probably find an application within the alloy industry [35].

Most of the research in this area focuses on analyzing the electrochemical behavior of lead deposition from eutectic solvents using pure reagents. The application of choline-chloride-based solvents in complex matrices such as secondary lead slag has been little explored.

In this study, we assess the feasibility of employing DES for the dissolution of lead from battery slag. The slag characterization was carried out in order to determine the concentration and chemical forms in which lead is found. To optimize this leaching process, the best experimental conditions were established such as sample type, temperature, time, and pulp concentration. We also conducted an electrowinning assay to evaluate this potential recovery path.

## 2. Results and Discussion

### 2.1. Sample Characterization

In this research, three different samples were investigated. All samples underwent the same treatment before leaching, involving hot water washes and roasting at 300 °C. Table 2 compares the physical and chemical properties of the samples with and without this treatment.

After pretreatment, all samples exhibited a significant decrease in pH values, likely due to the partial calcination of basic components added as fluxing and neutralizing agents during the pyrometallurgical process. Sample 1 presented the lowest pH value of 2.87 after thermal treatment. This was an expected result given that no neutralizing agent was added to this slag portion of the recycling plant. On both samples 2 and 3, sodium and calcium bicarbonates were incorporated as an environmental measure for neutralizing the slag of the stockpiles, resulting in elevated pH values. Since deep eutectic solvents have greater solubility for metal oxides in an acidic medium, it is expected that a higher quantity of lead will be leached when treating sample 1. In lower pH conditions, the concentration of H^+^ ions increases, and these protons can act as O^2-^ acceptors, breaking metal-oxide bonds and driving the solubilization process forward [22].

The conductivity of all samples was initially similar, around 94 mS.cm^−1^, and decreased to 24 mS.cm^−1^ after pretreatment. This decline in conductivity can be attributed to the hot deionized water washes, which removed a substantial portion of the soluble components present in the slag samples. Importantly, lead oxides and sulfates exhibit negligible or nonexistent solubility in water. This implies that the overall leaching process can be optimized, as the deep eutectic solvents will not become saturated with different cations apart from lead.

Sample 1 had the lowest moisture content as no liquid agents were added. Sample 2, being the freshest sample treated with lime milk, had the highest moisture content at 18.32%. Sample 3, also treated with lime milk, most likely lost water by drying in the pile exposed to ambient conditions during a six-month period.

In terms of ash content and loss for ignition analysis, all three slag samples were mainly constituted by inorganic and non-volatile components, accounting for over 80% of their composition. This was further verified through the elemental and compound analysis performed via X-ray Fluorescence and X-ray Diffraction.

The elemental and mineralogical analysis from Table 3 and Table 4 aligns with the typical composition for this type of slag, characterized by a CaO-FeO-SiO_2_ system, as reported in the literature [36].

While the metal of interest, lead, was found in percentages ranging from 1.9% to 5.4% (for treated samples), the most abundant element was iron with concentrations between 24.1% and 26.3%. Iron comes from ironstone, which is added during the smelting process to displace lead from sulfides, releasing it in a reduced state. Sulfur in the samples originates from the wasted PbSO_4_ and PbS electrodes as well as the remaining sulfuric acid from the used batteries (9.6–10.3%). Calcium and sodium are typically added to the process as fluxing agents in the form of CaO, NaCO_3_, or Na_2_B_4_O_7_, which serve to lower the melting temperature and transport unwanted metal oxides to the slag. Aluminum and silicon are components of sand, which are also incorporated into the mix in order to fix impurities in the calcium/sodium silicate matrix of the slag [10].

According to Zhang et al. (2012), aluminum and silicon oxides (Al_2_O_3_, SiO_2_) are insoluble in deep eutectic solvents, whereas calcium oxide barely has a solubility of 6 ppm on reline. These compounds are unlikely to interfere with the leaching process. Given that choline chloride systems are selective for metallic elements, it can be inferred that silicon and sulfur will not interfere with lead solubilization either. Iron oxides are the only compounds that may significantly impact the leaching of lead by also being solubilized in the deep eutectic solvents. In the case of reline, Fe_2_O_3_ has an experimental solubility of 49 ppm and Fe_3_O_4_ of 40 ppm. However, the solubility of lead oxide PbO_2_ is considerably higher at 9157 ppm [18]. It is worth mentioning that PbO and PbSO_4_ have proven to behave similarly to PbO_2_ [2].

Due to the substantial amount of amorphous material in the samples, a preliminary XRD analysis did not reveal any identifiable compounds. After calcinating the samples above 950 °C, specific minerals were identified and quantified, as listed in Table 4.

All samples presented hematite (Fe_2_O_3_) as its most abundant component. This result agrees with the one obtained by FRX analysis, where elevated concentrations of iron were demonstrated. Hematite, along with other iron oxides like maghemite, exhibits significant solubility on DES, suggesting a possible interference with lead leaching. However, iron may also be present in other forms when samples have not been calcinated. Wustite (FeO) and pyrrhotite (FeS) are some examples of other possible iron compounds that could be found on secondary lead slag [4].

Sulfur and sodium were also identified as abundant elements, mainly in the form of sulfates such as thenardite (Na_2_SO_4_) and anglesite (PbSO_4_). Lead was mostly found in the form of anglesite, fortunately, one of the most soluble forms on DES [2]. If lead is also present in the form of sulfate during leaching tests, elevated recovery results could be expected. Lastly, it can be inferred that the plagioclase groups found in the slag samples come from the sand incorporated into the mix during the pyrometallurgical processing, as discussed previously.

Full-scan ICP-MS analyses were conducted on TCLP extracts from the three untreated slag samples using the EPA 1311 procedure. This allowed us to compare the results with international regulations (CFR Title 40). Elements that leached at concentrations above 0.1 mg.L^−1^ are listed in Table 5.

Sample 3 had the highest sulfur concentration at 15,782.0 mg.L^−1^ but mobilized the least amount of metals. It presented almost a third part of calcium and much lower values of lead, magnesium, and manganese compared to the other samples. These results show that the slag oxidation contributed to the metal stabilization within the silicate matrix. However, this sample cannot be considered safe since, like the others, it surpasses the permissible limit for lead, which according to de CFR Title 40, is ≤5 mg.L^−1^. Sample 2 mobilized higher quantities of elements compatible with a basic pH medium, such as calcium, magnesium, and potassium, likely due to the fresh lime milk poured on the stockpile. Finally, sample 1 proved to be the most environmentally hazardous, with a lead concentration of 26.0 mg.L^−1^ in its leachate. It exceeds twice that of sample 2, triple that of sample 3, and five times the permissible limit of regulations. This can be explained by the affinity of lead towards acidic mediums. Despite being the worst possible source of contamination, sample 1 may be more suitable for the leaching process with deep eutectic solvents due to its abundant lead content and the excess of H^+^ ions.

Once again, the importance of decontaminating secondary slag waste has been demonstrated so that earthly resources are not threatened by the natural leaching of lead. The prior removal of lead is essential so that the battery recycling company complies with the regulations and permissible limits.

### 2.2. Conventional Leaching Assays

**Figure 1:**
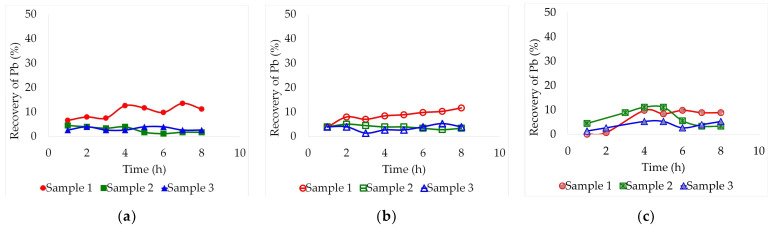
Leaching recovery of Pb (%) from the three different lead slag samples by lixiviation at 30 °C employing different deep eutectic solvents: (**a**) Ethaline, (**b**) Glyceline, (**c**) Reline. Pulp concentration = 2%.

**Figure 2:**
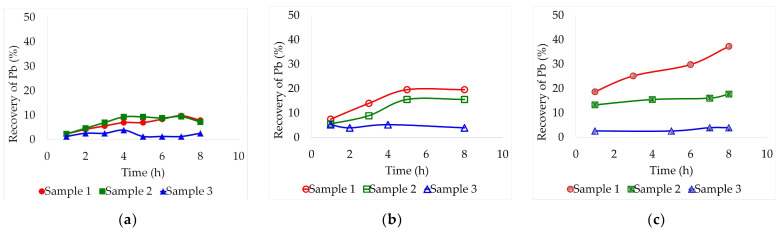
Leaching recovery of Pb (%) from the three different lead slag samples by lixiviation at 60 °C employing different deep eutectic solvents: (**a**) Ethaline, (**b**) Glyceline, (**c**) Reline. Pulp concentration = 2%.

**Figure 3:**
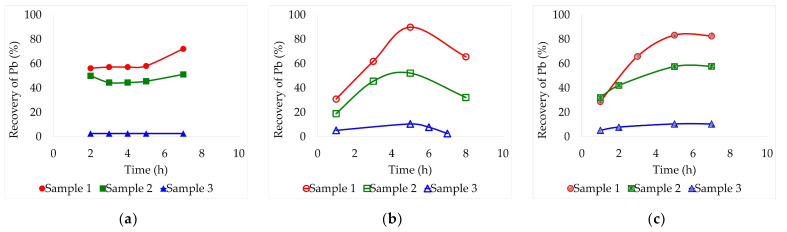
Leaching recovery of Pb (%) from the three different lead slag samples by lixiviation at 90 °C employing different deep eutectic solvents: (**a**) Ethaline, (**b**) Glyceline, (**c**) Reline. Pulp concentration = 2%.

Figure 1, Figure 2 and Figure 3 show the combined effect of temperature and lead leaching time from the three samples using three DES: ethaline, glyceline, and reline. These assays were carried out at three different temperatures (30, 60, and 90 °C) over an 8 h period. A consistent pulp concentration of 2% (for low viscosity) and magnetic stirring of 470 rpm were maintained constant for all tests. Aliquots were collected every hour to carry out the analysis of the dissolved lead by Atomic Absorption Spectrometry (AAS).

At 30 °C, lead leaching recovery was limited to a maximum of 14% due to insufficient heat and too much solvent viscosity. Within this range, variations were almost negligible. The process was not efficient at this temperature because there was not enough energy to break through the metal-oxygen or metal-sulphate bonds. In ethaline, Figure 1a, samples 2 and 3 maintained a constant leaching recovery between 1.1% and 4.4% without showing any upward trend. On the other hand, sample 1 started with a value of 6.5%, reaching a maximum of 13.5% after 7 h of leaching. In the case of glyceline, Figure 1b, the leaching recovery of samples 2 and 3 was similarly poor as in ethaline, remaining below 5.2%, but sample 1 presented a clear ascending trend throughout the assay, reaching a maximum yield of 11.6% after 8 h. Finally, using reline as a solvent, it can be seen in Figure 1c that the process did not present any clear trends. Unlike the other solvents, the leaching recovery on reline was slightly higher for sample 2, reaching 11.1% after just 4 h and decreasing again by the sixth hour. Sample 1 also rose at the fourth hour up to 9.8% and then remained constant until the end of the experiment. Sample 3 presented the highest resistance to leaching in every case. This may be due to the fact that the sample had a lower concentration of lead in relation to the other samples.

By carrying out the test with a temperature of 60 °C, lead leaching on sample 1 was favored by increasing its concentration on the deep eutectic solvents. Unfortunately, this increase could barely be evidenced for samples 2 and 3. There were also no significant improvements by employing ethaline as a solvent (Figure 2a). Sample 1 presented the highest recoveries, around 37.3% after 8 h in reline and 19.6% after 5 h in glyceline (Figure 2b,c). Sample 2 achieved maximum recoveries at the same times, 8 h in reline and 5 h in glyceline, 17.7% and 15.6%, respectively. At this temperature, reline proved to be the most effective deep eutectic solvent.

Finally, 90 °C results are presented in Figure 3 with the highest leaching recoveries achieved. Also, at this temperature, it was possible to better identify optimal lixiviation times. Sample 3 was an exception, as it did not benefit from the temperature increase (its recoveries remained the same as at 60 °C). With ethaline, Figure 3a, samples 1 and 2 exhibited similar behaviors, with recoveries remaining constant between 2 and 5 h, reaching their maximum at 7 h. These maximum recoveries were 72.2% and 51.2% each. With glyceline, Figure 3b, the best recovery for sample 1 was 90.0% at 5 h and for sample 2 it was 52.2% also at the fifth hour. Finally, on reline, Figure 3c, the highest leaching recoveries occurred after 5 h, being 83.4% for sample 1 and 57.8% for sample 2. Both samples presented similar behaviors on the three solvents reaching their maximums after the same time periods.

Ethaline as a solvent requires longer periods of time to dissolve lead compounds, even when subjected to a higher temperature. However, the optimal leaching times for glyceline and reline were clearly shortened by 2–3 h compared to the previous tests conducted at 60 °C.

The most optimal combination between the type of sample and the solvent was obtained with sample 1 and glyceline after 5 h of leaching at 90 °C. It can be inferred that leaching on sample 1 benefits from its low pH, given the larger presence of H^+^ protons. By comparing the behavior between sample 1 (pH = 2.87) and sample 3 (pH = 3.70), the influence of the pH on lead lixiviation was evident. As the pH increases, leaching is clearly diminished. In addition, it can be deduced that sample 3, when left to oxidize in the environment, was able to transform its chemical composition into more stable and difficult-to-leach components.

Previous studies have shown that as the temperature of the solvents increases, the pH also decreases. Also, temperature provides the necessary energy for the formation of metal-DES complexes and reduces the viscosity of the solvents, elevating the reaction rate as well as the mobility of the species [18].

Interestingly, some of the highest recoveries occurred in the middle of the test rather than at the end of it, specifically when using glyceline or reline. This phenomenon can be attributed to the loss of moisture in these deep eutectic solvents [37]. The water evaporation leads to a reduction in H^+^ ions, subsequently affecting the complexing capacity. Dehydration can serve as the starting point for a degradation process, causing a deviation from the eutectic point and a noticeable increase in the viscosity of the solvents.

To evaluate the influence of the pulp concentration on lead leaching, sample 1 was selected due to its promising recoveries in previous tests. The experiments were conducted using the three deep eutectic solvents, with a constant temperature of 90 °C and the optimal recovery times (7 h for ethaline and 5 h for both glyceline and reline). Lead leaching recovery was evaluated when modifying the slag concentration between 1, 2, and 5%, and the test results are displayed in Table 6.

In all instances, lead recovery through leaching was progressively enhanced with an increase in the pulp concentration. The highest recoveries were achieved by adding 5% of the slag to all three solvents (72.2% for ethaline, 95.0% for glyceline, and 84.4% for reline). However, it is important to note that as the amount of solid increases, the viscosity of the leachates also rises, posing challenges in the subsequent electrowinning test.

### 2.3. Electrowinning Assay (EW)

For the electrowinning test, 20 mL of a filtered lead leachate, dissolved in 80 mL of water was poured into the electrolytic cell. The leachate was prepared by conventional lixiviation based on the most effective determined conditions: sample 1, DES = glyceline, T = 90 °C, t = 5 h, stirring = 470 rpm, pulp concentration = 5%. In this case, a stainless-steel plate was used as a cathode and the leachate as the electrolyte source of lead cations. In order to negatively charge the stainless-steel electrode, graphite anodes were also immersed in the solution. Table 7 displays the ICP-MS analysis results for the 20:80 leachate-water solution.

The absence of iron in this leachate suggests that lead deposition will not be interfered with. Furthermore, the lead concentration obtained through ICP-MS (44.50 mg) agreed with the one obtained through atomic absorption (45.80 mg).

**Figure 4:**
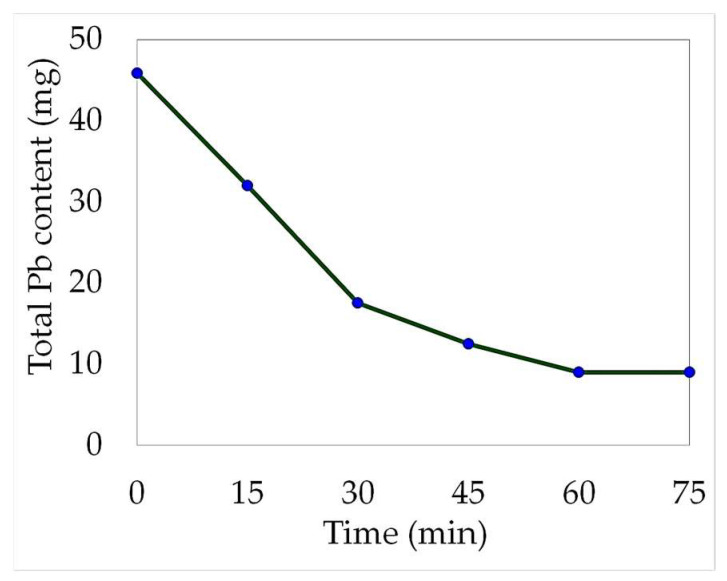
Lead content decrease of the 100 mL electrolyte (20:80 leachate-water solution) over time during the electrowinning development. (Electric current intensity = 0.1 A).

Figure 4 provides a graphical representation of the decrease in lead concentration over time during the electrowinning process. This reduction can be attributed to the lead recovery on the cathode or by the precipitation of the valuable metal towards the anode sludge. Between 60 and 75 min, the total lead content remained constant (9 mg). It is reasonable to assume that the maximum recovery had already been achieved by this point in time.

**Figure 5:**
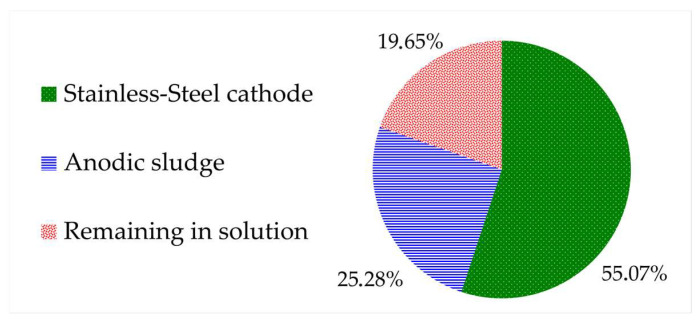
Summary of lead distribution throughout the electrowinning process.

The distribution of the total lead content throughout the electrowinning process was assessed. Within just 60 min, the lead concentration in the leachate decreased significantly, with only 19.7% of the total content remaining in the solution. This demonstrated that the electrodeposition process occurs rapidly under the current conditions employed. However, the total recovery of reduced lead at the cathode was only 55.1%, and a considerable amount precipitated to the anode sludge (25.3%). The electrowinning test results are displayed in Figure 5.

One of the reasons for the lead loss of the anode sludge may be the poor adherence in the cathode. To improve this adherence, various agents can be added to the electrolytic solution such as lead salts (nitrate or fluoroborate). These salts are usually added in small amounts to increase the concentration of the lead ions, improving nucleation mechanisms. Another option is the use of surfactants, such as polyethylene glycol (PEG), which can enhance the wetting of the cathode surface and improve the adhesion of the lead deposit. Surfactants also help to reduce the formation of dendritic growths when lead deposits tend to grow in a non-uniform manner [38]. Additionally, controlling the current density and temperature of the electrolyte might be necessary to find the highest current efficiency and to improve the overall electrowinning process. Once this electrolytic process is optimized, it could potentially serve as an attractive solution for the extraction of lead from leachates in deep eutectic solvents.

Only a few of the related studies, where deep eutectic solvents have been applied for the leaching of metals from complex matrices, have ventured into exploring subsequent recovery processes from the resulting solutions. This observation also emphasizes the importance of the present study. See Table 8.

Table 8 illustrates that deep eutectic solvents deliver notably high leaching recoveries for lead, even in complex samples. This is evident not only in secondary slag but also in diverse matrices such as flue dust, soil, and even polymetallic minerals. According to the findings presented by Argón-Tobar (2022), lead sulfides in polysulfide concentrates are inferred to contribute an additional soluble form to the list [37]. Only in the case of electric arc furnace dust, as investigated by both Abbott et al. (2009) and Bakkar et al. (2014), the obtained recoveries remained below 40%. Interestingly, in this case, the method appears to be more effective for zinc recovery than lead [35,39].

Abbott et al. (2009) conducted a recovery test based on lead cementation, which achieved a remarkable recovery rate of up to 97%. The effectiveness of this process suggests its potential applicability to automotive recycling slag. However, it is important to note that substantial quantities of powdered zinc or aluminum are necessary for this method. Additionally, it is worth considering that the presence of other metals in the leachate may influence the equilibrium constant of the process [39].

In the context of electrowinning or electrodeposition processes, the predominant focus of research has centered on assessing the purity and surface characteristics of the electrodeposits. Very little information regarding the quantification of the recovered metal has been found, a critical factor of significant industrial relevance. This study provides the initial estimate that approximately 55.1% is recoverable through electrowinning under the suggested conditions. Nevertheless, there is still a need for further investigations to optimize this process.

## 3. Materials and Methods

### 3.1. Sampling

Three samples of battery slags were provided from the stockpiles of a local automotive battery factory located in Quito, Ecuador. These samples (5 kg each) were transported to the laboratory for proper homogenization and size reduction.

Sample 1 corresponds to the slag that leaves the process directly to the pile without any type of treatment. Sample 2 was treated on-site with lime solution for neutralization, and Sample 3 had the same treatment as Sample 2 but was also left to dry and oxidize in the stockpile for a period of 6 months.

### 3.2. Sample Preparation

Initial sample preparation consisted of homogenization, crushing, drying, and grounding (<100 µm). Representative portions of 500 g were taken for the corresponding physical, chemical, and mineralogical analyses.

For the leaching assays, samples had to be washed with hot distilled water to minimize the presence of soluble species that could saturate the deep eutectic solvents. Roasting at 300 °C, below the melting point of lead, 327.4 °C [43], was intended for its transformation into oxides (PbO, PbO_2_) as well as sulfates (PbSO_4_). According to the literature, these are the most soluble forms of lead in deep eutectic solvents [2].

### 3.3. Sample Characterization

Water content was analyzed through the conventional stove drying method. Samples were dried in a stove for 2 h at 105 °C and the weight difference between container with wet sample and dry sample was determined using an analytical balance with a resolution of 0.01 g. Loss in ignition as well as the ash content were analyzed via calcination. Samples were placed in a muffle furnace for 3–6 h at 950 °C, and the weight difference between the tared crucible with the uncalcinated sample and the calcinated sample was determined using an analytical balance with a resolution of 0.0001 g. For pH and conductivity analysis, 2:1 extracts with deionized water were prepared by agitating for 1 h and centrifuging for 2 min at 1500 rpm. The measurements were made by immersing calibrated electrodes of a pH meter and a conductivity meter into the obtained extracts. It was important to perform such an analysis in order to determine the influence of these parameters on lead dissolution. Finally, density was measured by relating the weight of the samples with their occupied volume within a graduated cylinder.

Elemental analyses were conducted using X-ray Fluorescence (Bruker S8 Tiger unit with Spectra Plus program, Bruker Corporation, Madison, WI, USA, https://www.bruker.com/pt/products-and-solutions/elemental-analyzers/xrf-spectrometers/xrf-software.html, accessed on 27 January 2023) to determine the percentage of lead and the concentration of other abundant elements in the slag. Pressed tablets were prepared using 9 g of each sample with 1 g of binder. To identify possible crystalline phases, the X-ray Diffraction technique was applied (Bruker AXS D8 Advance). In this case, samples were prepared by regrinding small portions on an agate mortar. Qualitative and semi-quantitative analyses of the resulting diffractograms were carried out with the assistance of the EVA program and the TOPAS software (https://www.bruker.com/pt/products-and-solutions/diffractometers-and-x-ray-microscopes/x-ray-diffractometers/diffrac-suite-software/diffrac-topas.html, accessed on 27 January 2023). These tests aimed to determine the concentration and mineralogical phases containing Pb as well as any other elements that could possibly interfere with the leaching of lead.

In addition, full-scan analysis of TCLP extracts (Toxicity Characteristic Leaching Procedure, Agilent Technologies, Würzburg, Germany) was carried out by ICP-MS (Inductively Coupled Plasma Mass Spectrometry) (Agilent, 7850, Santa Clara, CA, USA) to assess the possible toxicity of the leachates when simulating the worst probable natural conditions. This analysis allowed us to determine the mobility of hazardous elements that were present in the slag samples. The leachate preparation procedure followed the EPA 1311 reference method.

### 3.4. Deep Eutectic Solvent Synthesis

Deep eutectic solvents were prepared based on choline chloride systems (98+%, Sigma Aldrich, St. Louis, MO, USA) in a 1:2 molar ratio with three hydrogen bond donors: Anhydrous urea for reline, Ethylene Glycol (99% Sigma Aldrich) for ethaline, and Glycerin (99.5% Fischer Scientific) for glyceline. The components were mixed in a beaker and continuously stirred at 80 °C for 2 h until transparent liquids were obtained. To enhance the oxidizing effect, 1 M Iodine (Baker, New York, NY, USA) was added to the solvents. Jenkin et al. (2016) proved that the use of this oxidizing agent can selectively increase the recovery of certain metals [44].

### 3.5. Leaching Assays

A weighted amount of sample was added to each deep eutectic solvent container. Then, these flasks were placed in water baths with magnetic stirring at 470 rpm. After the required time had elapsed, aliquots were extracted to measure the lead concentration through atomic absorption (Perkin Elmer AA 300, Yokohama, Japan). To optimize the leaching process, experiments were repeated at varying temperatures, time periods, and pulp concentrations.

The dissolution of metals in DES often involves complex electrochemical processes, and various intermediate species can be involved. The following is a simplified chemical reaction process for lead dissolution in choline chloride-based deep eutectic solvents:

(1) $$2{\mathrm{PbO}}_{\left(s\right)}+4{H^{+}}_{\left(\mathrm{aq}\right)}\to 2{{\mathrm{Pb}}^{2+}}_{\left(\mathrm{aq}\right)}+2H_{2}O;\;{\mathrm{PbSO}}_{4\left(s\right)}+2{H^{+}}_{\left(\mathrm{aq}\right)}\;\to \;2{{\mathrm{Pb}}^{2+}}_{\left(\mathrm{aq}\right)}+H_{2}{\mathrm{SO}}_{4}$$

(2) $${{\mathrm{Pb}}^{2+}}_{\left(\mathrm{aq}\right)}+4{{\mathrm{Cl}}^{-}}_{\left(\mathrm{aq}\right)\;}⇌{{\left[{\mathrm{PbCl}}_{4}\right]}^{-2}}_{\left(\mathrm{aq}\right)}$$

The dissociation of oxides and sulfates are not generally spontaneous reactions, which is why they require an excess of hydronium ions (H^+^) facilitated by the hydrogen bond donor (HBD) in order to occur (Equation (1)). Lead cations engage with chloride radicals, giving rise to the formation of lead tetrachloride ions (Equation (2)). The obtained lead tetrachloride ions are soluble and very likely to interact with other protonated species (H^+^). According to Liao et al. (2016), PbO_2_ and PbSO_4_ exhibit the same behavior as PbO, indicating that Pb^2+^ ions are formed regardless of what Pb compound is introduced [2].

While deep eutectic solvents are generally recognized as environmentally friendly, it is important to note that when the hydrogen bond donor (HBD) or hydrogen bond acceptor (HBA) components are volatile, they can potentially emit toxic substances and contribute to air pollution [31]. In the context of this study, the organic compounds employed are non-volatile, indicating that the solvent synthesis process is not likely to be environmentally hazardous.

Nevertheless, it is crucial to consider that during the leaching procedure, the presence of sulfate ions and an excess of protons [H^+^] in the system can lead to the formation of sulfuric acid, as illustrated in Equation (2). This may impact the quality of the solvent, potentially rendering it unsuitable for reuse and necessitating neutralization as an environmental control measure. Therefore, it is imperative to address and thoroughly investigate this factor, while also assessing the condition of the residual solvent after the process.

### 3.6. Electrowinning Assay

For the electrowinning test a volume of 100 mL of lead leachate was prepared based on the best previously determined conditions of time, temperature, type of sample, pulp concentration, and DES. Vacuum filtration was required in order to remove solids (0.45 µm). Then, taking advantage of its solubility, a 20:80 leachate-water solution was prepared and poured into an electrolytic cell. The objective of diluting the leachate in water was to reduce its viscosity and to favor the electrowinning process. Inside the cell, a stainless-steel cathode was placed between two carbon graphite anodes, and an electric current of 4.32 V was allowed to pass between the electrodes, resulting in an amperage of 0.1 A. The electric current condition was the same as the one employed on the typical electrowinning route for lead from hydrofluorosilicic acid [12].

Every 15 min of the electrowinning process, aliquots of the leachate were collected in order to measure the loss in lead concentration by atomic absorption spectrometry. The lead content was also measured in the sludge residue that precipitated at the bottom of the electrolytic cell. A series of calculations were used to account for the distribution of lead throughout the process and also to determine the final recovery percentage.

The potential half-reactions involved in the electrolytic process are as follows:

(3) $$\mathrm{Dissociation}:\;{{\left[{\mathrm{PbCl}}_{4}\right]}^{-2}}_{\left(\mathrm{aq}\right)}\;⇌{{\;\mathrm{Pb}}^{2+}}_{\left(\mathrm{aq}\right)}+4{{\mathrm{Cl}}^{-}}_{\left(\mathrm{aq}\right)}$$

(4) $$\mathrm{Cathode}:\;{{\mathrm{Pb}}^{2+}}_{\left(\mathrm{aq}\right)}+2{e^{-}}_{\left(\mathrm{aq}\right)}\;\to {{\;\mathrm{Pb}}^{0}}_{\left(s\right)}$$

(5) $$\mathrm{Anode}:\;{{\mathrm{Pb}}^{2+}}_{\left(\mathrm{aq}\right)}\;\to {{\;\mathrm{Pb}}^{4+}}_{\left(\mathrm{aq}\right)}+2{e^{-}}_{\left(\mathrm{aq}\right)}$$

## 4. Conclusions and Future Perspectives

The present study proved the feasibility of applying deep eutectic solvents as leaching agents for the removal and recovery of lead from the slag generated in the automotive battery recycling process.

It was necessary to carry out the previous characterization of the treated slag where lead was found in percentages between 1.91% and 5.36% in its most probable form of anglesite (PbSO_4_). This compound had an appropriate solubility for the proposed treatment as found in previous studies and later demonstrated experimentally. The primary interferent identified in the samples was iron, with concentrations ranging from 24.10% to 26.33%, mainly in the form of oxides. These oxides have considerable solubility with respect to DES, but fortunately, they are not comparable to that of lead.

By means of TCLP extraction, it was demonstrated that none of the samples (7.0–26.0 mg.L^−1^ Pb) complies with the permissible limits of the regulations (≤5 mg.L^−1^ Pb) for mobilizing large amounts of lead. This highlighted the importance of decontaminating the slag and recovering the valuable metal.

For the leaching process, five operational variables were evaluated: type of sample, type of DES (varying the hydrogen bond donor), temperature (30, 60, 90 °C), time (1–8 h), and pulp concentration (1, 2, 5%). Sample 1 showed higher lixiviation recoveries due to its low pH, proving that by increasing the H^+^ concentration, the solubilization process is favored. Glyceline was chosen as the best leaching agent after 5 h of agitation and a temperature of 90 °C. At this temperature, the solution viscosity was decreased and the reaction rate elevated. By preparing the solution under the aforementioned conditions with a pulp concentration of 5%, it was possible to achieve a leaching yield of 95.0%.

Through an electrowinning test, the ability to recover lead from the leachate was demonstrated with an efficiency of 55.1%.

Regarding future developments in this field of research, there is a wide variety of different HBDs or mixtures of them that could also be tested. Additionally, various operational parameters offer avenues for further investigation. These may include adjustments to solvent pH, variation in stirring speed, the selection of different oxidizing agents, and the optimization of surface complexation through the addition of coordinating ligands. In the case of electrowinning, it is advisable to conduct a comprehensive cathodic sweep using cyclic voltammetry to pinpoint the precise deposition peak. This step can assist in determining the ideal cell voltage and current density. Exchanging the stainless-steel cathode for one of greater reducing potential could also improve the electrowinning process. Ensuring that the electrode’s surface area matches the quantity of lead in the electrolyte is crucial. Finally, other forms of recovery could be explored, such as cementation. However, for any process, it is important to consider ecological and economic factors such as energy consumption and reagents expenses.

Assessing the condition of the residual DES following the electrowinning process could serve as an intriguing research proposal. This investigation would aim to ascertain the potential for its reuse or explore alternative applications based on its physical and chemical properties.

Ideally, the recovered lead should be reintroduced into the production process of automotive battery electrodes. This approach aligns with the principles of the circular economy, which is increasingly recognized as a sustainable and responsible practice. But even if the secondary slag residue does not go through a subsequent recovery process, the ability to decontaminate it has been demonstrated for compliance with regulations. The proposed methodology approaches to an environmentally friendly alternative.

## Figures and Tables

**Table 1:** Physical properties of DES.

DES	Viscosity (cP)	Density ρ (g.cm^−3^)	pH	Conductivity (k, mS.cm^−1^)	Freezing Point (°C)	Reference
Ethaline (ChCl-Ethylene Glycol)	52 (at 20 °C)	1.14 (at 20 °C)	4.676	6.17 (at 20 °C)	−66	[18,20,25,26]
Glyceline (ChCl-Glycerol)	376 (at 20 °C)	1.19 (at 20 °C)	7.543	2.03 (at 20 °C)	−40	[18,20,25,26]
Reline (ChCl-Urea)	750 (at 25 °C)	1.24 (at 25 °C)	10.387	0.20 (at 40 °C)	1	[18,19,27,28]

**Table 2:** Characterization of the slag samples before and after pre-treatment (hot water washes and 300 °C roasting).

Characterization	Sample 1		Sample 2		Sample 3	
	Before	After	Before	After	Before	After
pH	8.53	2.87	9.85	4.81	9.96	3.70
Conductivity (mS.cm^−1^)	94.57	23.43	94.17	26.67	93.63	24.00
Moisture Content (%)	3.49		18.32		9.28	
Ash Content (%)	95.34		81.52		88.79	
Loss in ignition (%)	0.043		0.063		0.038	
Density (Kg.m^−3^)	1251.6	1355.8	990.7	1356.8	1127.0	1218.9

**Table 3:** X-ray Fluorescence analysis of the slag samples before and after pre-treatment (hot water washes and 300 °C roasting).

Element	Sample 1		Sample 2		Sample 3	
	Before (%)	After (%)	Before (%)	After (%)	Before (%)	After (%)
Fe	25.1	26.3	23.7	24.6	21.6	24.1
S	11.1	9.6	9.7	9.7	10.9	10.3
Na	10.8	11.7	10.5	11.7	11.9	15.3
Pb	3.6	5.4	3.5	4.5	0.9	1.9
Si	1.9	2.1	2.4	2.4	1.8	2.1
Al	0.6	0.8	0.9	0.9	0.7	0.8
Ca	0.5	0.6	0.5	0.6	0.4	0.5
Ba	0.4	0.3	0.4	0.4	0.4	0.4
Sn	0.3	0.3	0.3	0.4	0.2	0.2

**Table 4:** X-ray diffraction analysis of the slag samples calcinated over 950 °C.

Name	Compound	Sample 1 (%)	Sample 2 (%)	Sample 3 (%)
Hematite	Fe_2_O_3_	58	53	60
Thenardite	Na_2_SO_4_	30	36	36
Anglesite	PbSO_4_	10	5	<1
Maghemite	Fe_2_O_3_	<1	4	2
Plagioclase Group	(Na,Ca)Al(Si,Al)Si_2_O_8_	2	2	2

**Table 5:** ICP-MS Elemental Analysis of TCLP leachates of the untreated slag samples.

Element	Sample 1 (mg.L^−1^)	Sample 2 (mg.L^−1^)	Sample 3 (mg.L^−1^)
S	4217.0 *	4094.0 *	15,782.0 *
Ca	185.0	189.0	69.0
Fe	136.0	82.0	<0.1
Pb	26.0	14.0	7.0
Si	24.0	44.0	8.2
K	22.0	26.0	19.0
Mg	10.0	15.0	0.9
Mn	9.8	5.6	0.7
B	2.2	2.8	0.3
Zn	2.6	1.6	<0.1
Ni	1.3	1.5	<0.1

* The values corresponding to sulfur are considered referential since they exceed the application range of the method.

**Table 6:** Leaching recovery of Pb (%) from sample 1 by leaching at 90 °C and varying the pulp concentration (1%, 2%, 5%) on the three deep eutectic solvents: Ethaline, Glyceline, and Reline.

DES	Temperature (°C)	Lixiviation Time (h)	Percentage of Solids (%)	Recovery of Pb (%)
Ethaline	90	7	1	52.4
			2	66.6
			5	72.2
Glyceline	90	5	1	67.2
			2	90.0
			5	95.0
Reline	90	5	1	75.6
			2	83.4
			5	84.4

**Table 7:** ICP-MS Elemental analysis of the 20:80 leachate-water solution.

Element	Concentration (mg)
Ba	0.75
Cu	3.00
Mn	1.00
Pb	44.50
Sb	0.20

**Table 8:** Comparison between obtained results and those reported in the literature.

Sample Material	Leached Compound	Deep Eutectic Solvent	Leaching Yield	Recovery Method	Metal Recovery	Reference
Secondary Lead Slag	Mainly PbSO_4_	ChCl-Glycerol	95% Pb	Electrowinning	55.1% Pb	This study
Electric Arc Furnace Dust	ZnO, PbO	ChCl-Urea, ChCl-EG	±35% Pb, ±70% Zn	Electrolysis (Zn), Cementation (Pb)	(63–97)% Pb	[39]
Electric Arc Furnace Dust	ZnO, PbO	ChCl- Urea	39% Pb, 60% Zn	Electrowinning	-	[35]
Cupola Furnace Dust	ZnO, PbO	ChCl-Urea, ChCl-EG	38% Zn	Electrodeposition	-	[40]
Soil	Pb	ChCl-Fructose, ChCl-Sucrose, ChCl-EG, ChCl-Glycerol	>72%	-	-	[41]
Zinc Flue Dust	Fe, Zn, Pb, Cu, In, Sn	ChCl-Urea, ChCl-EG, ChCl-Oxalic Acid	93% Pb, >80% others	-	-	[42]
Polysulfide Mineral Concentrate	CuFeS_2_, FeS_2_ (Zn,Fe)S, PbS, PbSO_4_,	ChCl-EG, ChCl-Glycerol, ChCl-Urea	(93–96)% Pb (24–30)% Cu (65–85)% Zn 22% Fe	-	-	[37]

## Data Availability

Data are contained within the article.
